# Dataset and methodological framework for land-use change scenario modelling in Uruguay using the Land Change Modeler

**DOI:** 10.1016/j.dib.2026.113287

**Published:** 2026-09-28

**Authors:** Melanie Schweikart, Leonardo R. Ramírez, Ina Säumel

**Affiliations:** a Department of Geography, Humboldt-Universität zu Berlin, Unter den Linden 6, Berlin, 10099, Germany; b Integrative Research Institute THESys Transformation of Human-Environment-Systems, Humboldt-Universität zu Berlin, Unter den Linden 6, Berlin, 10099, Germany; c Thaer-Institute of Agricultural and Horticultural Sciences, Humboldt-Universität zu Berlin, Unter den Linden 6, Berlin, 10099, Germany

**Keywords:** Multi-layer perceptron, Markov chain, Grassland conservation, Spatial planning, Transition potential, TerrSet, Agroecological transitions, Remote sensing

## Abstract

This article presents a spatial dataset and methodological workflow for simulating land-system dynamics in Uruguay using the Land Change Modeler implemented in TerrSet v.2020. The dataset combines harmonized land-cover maps for 2000, 2015, and a validation dataset for 2020 with explanatory variables, transition probability matrices, and scenario-based simulations developed through a multi-layer perceptron–Markov chain approach. A key feature of the dataset is the harmonization of heterogeneous national land-use datasets into a consistent modelling framework, combined with a transparent workflow for explanatory variable selection, transition modelling, and scenario parameterization. The dataset provides the final scenario outputs together with the transition probability matrices, conservation constraint and incentive layers, validation outputs, and the methodological workflow required to reproduce the analyses and adaptation of the workflow to other regions. Three land-use policy pathways were implemented: Business-as-Usual (BAU), Multifunctional Landscape (MFL), and Grassland Conservation (GC). The dataset includes high-resolution raster layers, conservation constraint and incentive maps, explanatory variable influence assessments, and model validation outputs supporting reproducible spatial analyses and territorial planning applications. These data complement the associated Land Use Policy article, Balancing Growth and Conservation: Land-Use Policy Scenarios for Uruguay’s Grasslands [1], by providing the underlying spatial datasets, methodological details, and validation framework required for reproducibility and reuse. The resources facilitate further research on grassland conservation, agroecological transitions, and scenario-based environmental assessment.

Specifications Table

**Table:** 

Subject	Earth & Environmental Sciences
Specific subject area	Land-use change modelling; conservation planning; spatial simulation; territorial planning
Type of data	Raster datasets (GeoTIFF); tabular data including transition probability matrices (CSV)
Data collection	Land-use maps for 2000, 2015, and 2020 were obtained from Uruguay’s Ministry of Housing, Land Management and Environment (MVOTMA/Ministry of Environment) and harmonized to a common nine-class land-use classification. Vector datasets were converted to 30-m raster format, reprojected (WGS84/UTM 21S), resampled where required, and combined with 20 environmental, infrastructural, climatic, and socio-economic explanatory variables using QGIS v3.22.4. Land-use transitions were modelled in TerrSet v2020 using the Land Change Modeler (LCM) with a Multi-Layer Perceptron–Markov Chain approach after variable screening based on correlation analysis and backward stepwise elimination.
Data source location	Institution: Ministry of Housing, Land Management and Environment (MVOTMA; now Ministry of Environment), Uruguay. City/Town/Region: Nationwide coverage. Country: Uruguay. Spatial extent: Entire national territory of Uruguay (approximately 30°–35° S, 53°–58° W).
Data accessibility	The data supporting this article are included within the article and its tables and figures. The raw data are accessible at the Humboldt-Universität zu Berlin Institutional Repository (edoc-Server) https://doi.org/10.18452/37602. To access the compressed file in window environments use the “7-Zip” software available at https://www.7-zip.org/.
Related research article	M. Schweikart, L.R. Ramírez, I. Säumel, Balancing growth and conservation: Land-use policy scenarios for Uruguay's grasslands. Land Use Policy 169 (2026) 108,110. https://doi.org/10.1016/j.landusepol.2026.108110

## 1 Value of the Data

• These data enable high-resolution analyses of land-use change in Uruguay, providing a robust basis for understanding spatial dynamics over time.
• They support the development of scenario-based policy recommendations for conservation, sustainable land management, and territorial planning.
• The dataset facilitates the replication and adaptation of the presented methods in other regions that face comparable land-use challenges.
• It offers detailed methodological insights for researchers applying the Land Change Modeler in land-use planning and related environmental assessments.
• Unlike most published land-use scenario datasets, this resource documents the complete modelling workflow, explanatory variable selection procedure, transition probability matrices, validation outputs, and policy-specific parameterization, enabling transparent reproduction of the modelling approach.

## 2 Background

Land-use change modelling is widely used to explore the consequences of alternative land management strategies and to support spatial planning by integrating historical land-use dynamics with environmental and socio-economic drivers [1,2]. The Land Change Modeler (LCM) implemented in TerrSet combines Multi-Layer Perceptron (MLP) neural networks with Markov chain analysis to simulate spatially explicit land-use transitions and has been successfully applied to a wide range of land-use change studies worldwide [[3], [4], [5], [6], [7]].

Uruguay's temperate grasslands have experienced substantial land-use change during recent decades, mainly driven by agricultural intensification and the expansion of commercial forestry, resulting in increasing pressure on native grassland ecosystems and biodiversity [8,9]. Reliable spatial datasets are therefore required to evaluate alternative policy pathways and quantify their potential impacts on future land-use patterns.

This dataset was developed to provide the complete spatial data and methodological workflow underlying the accompanying Land Use Policy article [10]. It integrates harmonized national land-use maps (2000, 2015 and 2020), explanatory variables, transition probability matrices, model calibration and validation outputs, and policy-specific constraint and incentive layers used to simulate three land-use scenarios: Business-as-Usual (BAU), Multifunctional Landscape (MFL), and Grassland Conservation (GC). In contrast to many published scenario studies that report only final model outputs, this dataset includes the intermediate products required to reproduce, evaluate and adapt the modelling workflow using the Land Change Modeler [3,11].

By making these datasets and methodological components publicly available, this article supports reproducible land-use change modelling and facilitates the application of comparable workflows in other grassland regions facing similar land-use pressures.

## 3 Data Description

This dataset consists of: 1) Refined historical land-use maps (2000, 2015, and validation dataset for 2020) in raster format. 2) Transition probability matrices for land-use change modeling in tabular data (2015 to 2020 and 2015 to 2030). 3) 20 explanatory variables used in land-use transition simulations in raster format (available upon request). 4) Delineating sensitive areas for conservation buffer scenarios in raster format (incentive and constraint maps). 5) Accuracy assessment of classification maps (2000, 2015, 2020) in tabular data. 6) Model validation outputs for BAU scenario prediction for 2020 in raster format. 7) Model outputs for BAU, MFL, GC scenario prediction for 2030 in raster format.

### 3.1 Historical transitions in land use/cover

Table 1 presents the historical trends in land use and land cover change from the year 2000 to 2015. Crops & artificial grasslands and timber plantations show the highest net change per class at 5.7 % and 1.52 %, respectively, primarily influenced by the conversion of near-natural grassland. Only the transitions represented by the white cells were modelled, whereas transitions shown in grey were excluded (Table 1). For cropland expansion, the model included transitions from near-natural grassland, aquatic ecosystems, and shrubs. Near-natural grassland's net contribution significantly surpasses others, marking cropland change as the most dominant. While changes from aquatic ecosystems and shrubs to crops & artificial grasslands are minor compared to near-natural grassland, we included them to capture nuances, considering aquatic ecosystems (flooded areas often converted to cropland). Despite their minor contributions, aquatic ecosystems and shrubs add to the overall expansion of crops & artificial grasslands, making it the most relevant transition. For the second-largest expansion, timber plantation, the net contribution from aquatic ecosystems and shrubs is zero, indicating limited relevance in our data. Native forest experiences some gain but shows an overall negative net change. Although net contributions from areas undergoing natural succession (aquatic ecosystems, shrubs, and near-natural grassland) are negative, detailed change maps confirm ongoing transitions to native forests (Table 1).

**Table 1:** Gains, losses, and net changes by land-use classes (%) and net contributions to transitions between 2000 and 2015 (%).

Land-use classes	Loss (%)	Gain (%)	Net Change (%)	Net Contribution (%)									
				AE		NF	S	Hc	NNG	BS	U	CAG	TP
AE	−0.15	0.24	0.09	0.00		*−0.03	−0.01	0.00	−0.08	0.00	0.00	**0.02	0.00
NF	−0.62	0.28	−0.34	0.03		0.00	0.19	0.00	0.03	0.00	0.00	0.05	0.04
S	−0.24	0.28	0.03	0.01		*−0.19	0.00	0.00	−0.02	0.00	0.00	**0.16	0.00
Hc	−0.05	0.2	0.15	0.00		0.00	0.00	0.00	−0.16	0.00	0.00	0.02	0.00
NNG	−7.69	0.53	−7.16	0.08		*−0.03	0.02	0.16	0.00	0.00	0.02	**5.46	**1.46
BS	−0.02	0.02	0.00	0.00		0.00	0.00	0.00	0.00	0.00	0.00	0.00	0.00
U	−0.01	0.04	0.03	0.00		0.00	0.00	0.00	−0.02	0.00	0.00	0.00	0.00
CAG	−0.29	5.99	5.7	−0.02		−0.05	−0.16	−0.02	−5.46	0	0	0	0.01
TP	−0.1	1.62	1.52	0		−0.04	−0.01	0	−1.46	0	0	−0.01	0

***Note:*** White cells represent modelled transitions, whereas grey cells indicate transitions excluded from modelling (*native forest succession; **cropland expansion; ***timber expansion). Abbreviations: Aquatic ecosystems (AE); native forests (NF); shrubs (S); horticulture (Hc); near-natural grasslands (NNG); bare soils (BS); urban (U); crops & artificial grasslands (CAG); timber plantations (TP).

### 3.2 Examining omitted and included explanatory variables

Variable selection was based on the backward stepwise variable elimination procedure implemented in the Land Change Modeler, systematically removing variables with minimal influence on model performance. Variables without explanatory power were excluded to reduce the risk of overfitting and improve model parsimony. In addition, highly correlated variables (Pearson r > 0.8) were removed to reduce redundancy. Variable importance was assessed by forcing individual explanatory variables to remain constant while evaluating changes in model performance. To account for potential variability caused by random weight initialization, the five most influential variables were selected for detailed interpretation (Fig. 1, Fig. 2).

**Fig. 1:**
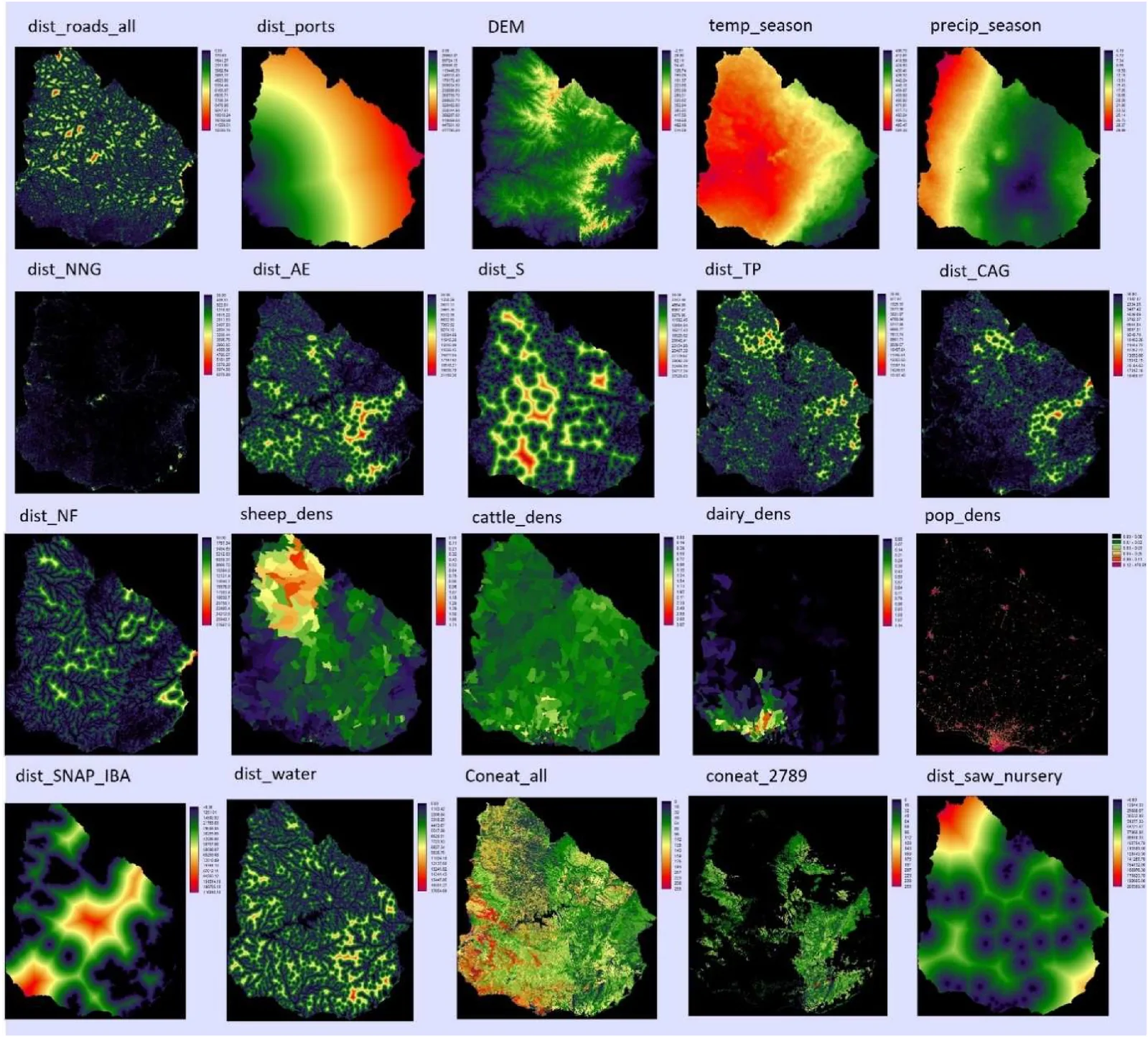
Spatial value distribution for the 20 explanatories variables used in transition models. All variables with “dis” in the name show high values in meters. Red indicates high distances, yellow medium, and blue/ green low distances. Temp_season and percip_season describe seasonality of temperature and precipitation. High to low values range from red to blue, indicating a higher variability over the year for the standard deviation based on monthly means. DEM show digital elevation. Coneat_all and coneat_2789 show the Uruguayan soil productivity index ranging low cost for land from blue to red for high costs and productivity for land. The three livestock variables show sheep, cattle and dairy cows per hectare. Population density is shown per pixel in pop_density.

**Fig. 2:**
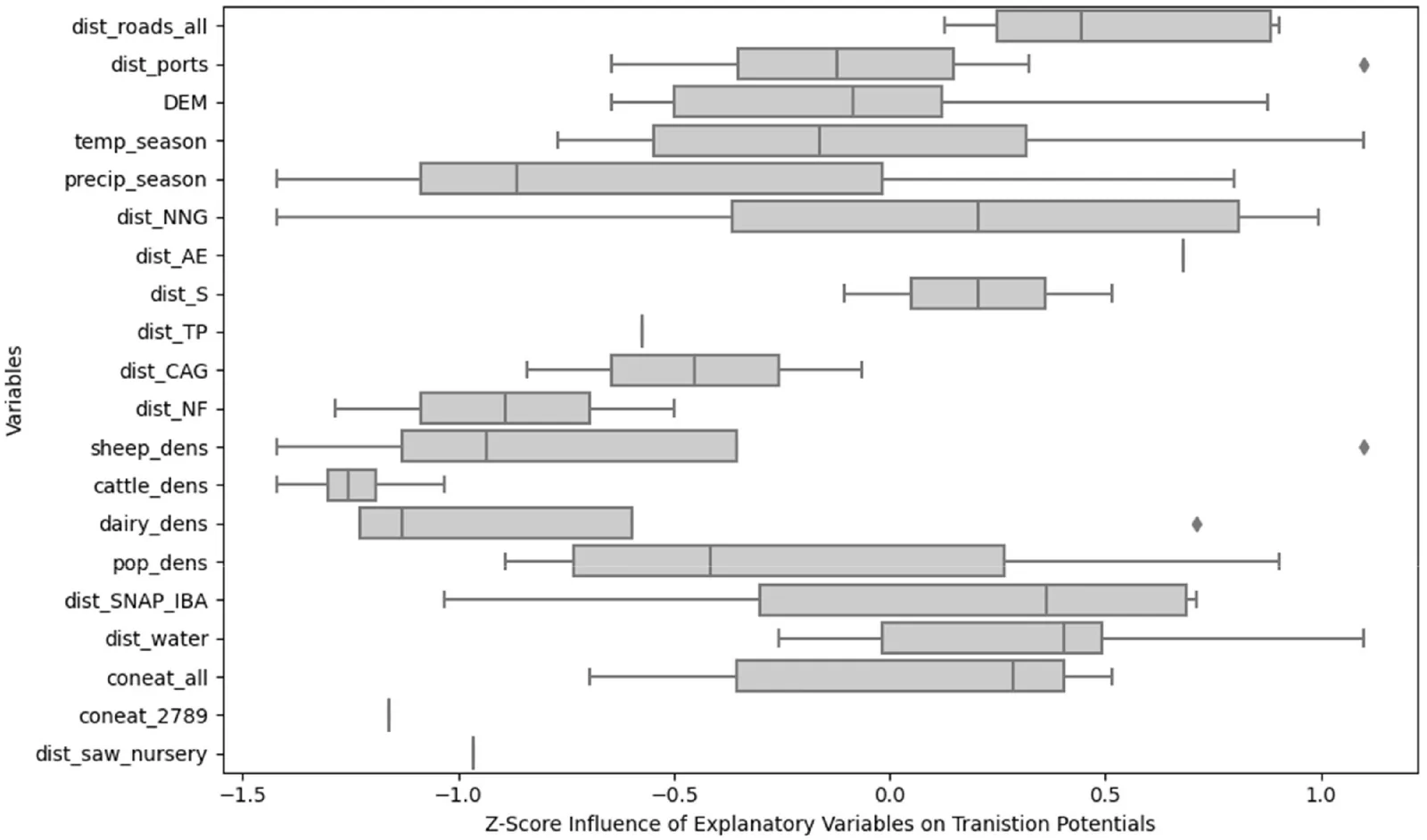
Influence of explanatory variables among all transition potential models. The values represent Z-scores. Positive values indicate values above the mean, suggesting low influence on the model, while negative Z-scores indicate values below the mean, signifying high influence.

The variable *dist_roads_big*, focusing on primary and secondary roads, was removed as *dist_roads_all*, which considers the entire road network, had a higher impact but still relatively low influence on the model (Fig. 1). *Dist_U* was also removed as it showed no relevance for any model. Dist_modis_fire, although potentially relevant for all transition models, did not prove to be so. Models improved after removing slope percentage. *DEM*, representing a similar driver for change, had a higher influence on the model and was considered.

*Pop_dens, DEM, dist_ports, temp_season, distNF*, and *coneat_all* emerged as the top five influential variables across three to two models. Contrary to expectations, *coneat_all, dist_roads_all*, and the distance to land-uses from 2000 did not exhibit overall high explanatory power, as anticipated based on their importance described in chapter *Details on Explanatory Variables* (below). *Coneat_2789* and *dist_saw_nursery* had a notable impact but were solely used for the timber expansion model [10].

Fig. 2 presents the distribution of variable influence across all explanatory variables as boxplots of the calculated Z-scores. The importance values are assigned from one to the number of included variables, ranging between 12 and 14. Z-scores summarize each importance value based on its deviation from the mean and standard deviation. Positive Z-scores indicate values above the mean, suggesting low influence, while negative Z-scores indicate values below the mean, signifying high influence.

### 3.3 Land-use policy prediction

The transition probability matrix for the BAU prediction for 2020 is presented in Table 2.

**Table 2:** Transition probability matrix with probabilities for BAU 2020 without adaptation. Rows represent 2015 land-use classes, changing based on probabilities in cell values to corresponding classes in 2020. Light grey cells show non-modelled transitions, maintaining 2015 persistence.

2020										
	Land-use classes	AE	NF	S	Hc	NNG	BS	U	CAG	TP
2015	AE	0.9787	0.0086	0.0002	0.0000	0.0064	0.0008	0.0000	0.0050	0.0002
	NF	0.0111	0.9187	0.0305	0.0004	0.0262	0.0003	0.0002	0.0057	0.0068
	S	0.0048	0.0118	0.8835	0.0002	0.0134	0.0001	0.0002	0.0787	0.0074
	Hc	0.0002	0.0008	0.0004	0.9703	0.0039	0.0000	0.0013	0.0221	0.0010
	NNG	0.0009	0.0013	0.0003	0.0013	0.9419	0.0001	0.0002	0.0427	0.0114
	BS	0.0162	0.0070	0.0029	0.0017	0.0191	0.9396	0.0024	0.0027	0.0088
	U	0.0002	0.0006	0.0002	0.0016	0.0044	0.0005	0.9899	0.0017	0.0008
	CAG	0.0004	0.0004	0.0003	0.0006	0.0070	0.0000	0.0001	0.9898	0.0013
	TP	0.0004	0.0025	0.0011	0.0005	0.0075	0.0002	0.0010	0.0040	0.9828

***Note:*** Aquatic ecosystems (AE); native forests (NF); shrubs (S); horticulture (Hc); near-natural grasslands (NNG); bare soils (BS); urban (U); crops & artificial grassland (CAG); timber plantation (TP).

### 3.4 Validation and Scenario Prediction Comparison

The predicted land-use areas for the BAU scenario in 2020 are compared with the validation map in Table 3. Table 4 compares the predicted land-use areas under the GC, MFL, and BAU scenarios within the old-growth grassland zone.

**Table 3:** Area share (km²) of land-use classes in the business-as-usual prediction map (2020), the validation map (2020) and area differences in reference to the validation map 2020.

Land-use classes	BAU prediction 2020	Validation map 2020	Area difference of validation map - prediction map	Proportion of differences to total area
	(km²)	(km²)	(km²)	(%)
Aquatic Ecosystems	6582	6075	−508	0
Native Forest	6676	8087	1411	1
Shrubs	1967	1570	−397	0
Horticulture	1898	4178	2279	1
Near-Natural Grassland	100,120	89,186	−10,934	−6
Bare Soils	328	231	−97	0
Urban	1344	1537	194	0
Crops & Artificial Grassland	46,359	54,150	7791	4
Timber Plantation	10,510	10,771	261	0

**Table 4:** Land-use areas and net area changes along the grassland conservation (GC), the multifunctional landscape (MFL), and the business-as-usual (BAU) scenario prediction within the old-growth Grassland zone. The old-growth grassland zone covers 17 % of Uruguay. NAC: net area change (land-use in larger protection area – land-use smaller protection area) / total area within (old-growth grassland zone)).

Within old-growth grassland zone					
Land-use classes	GC (km²)	MFL (km²)	NAC GC to MFL (%)	BAU (km²)	NAC GC to BAU (%)
Aquatic Ecosystems	220	220	0	194	12
Native Forest	876	876	0	876	0
Shrubs	247	237	4	237	4
Horticulture	80	80	0	80	0
Near-Natural Grassland	27,349	27,319	0	27,292	0
Bare Soils	11	11	0	11	0
Urban	18	18	0	18	0
Crops & Artificial Grasslands	2171	2181	0	2225	−2
Timber Plantation	182	212	−16	223	−22

## 4 Experimental Design, Materials and Methods

### 4.1 Land-use data collection & processing

This section describes the land-use datasets obtained from Uruguay’s Ministry of Housing, Land Management and Environment, the reclassification into nine land-use classes, the accuracy assessment using stratified random sampling and confusion matrices, and the harmonization of historical datasets using pixel masking based on the 2020 classification. The spatial distribution of predicted land-use classes under the BAU scenario for 2020 is compared with the corresponding validation map in Fig. 3.

**Fig. 3:**
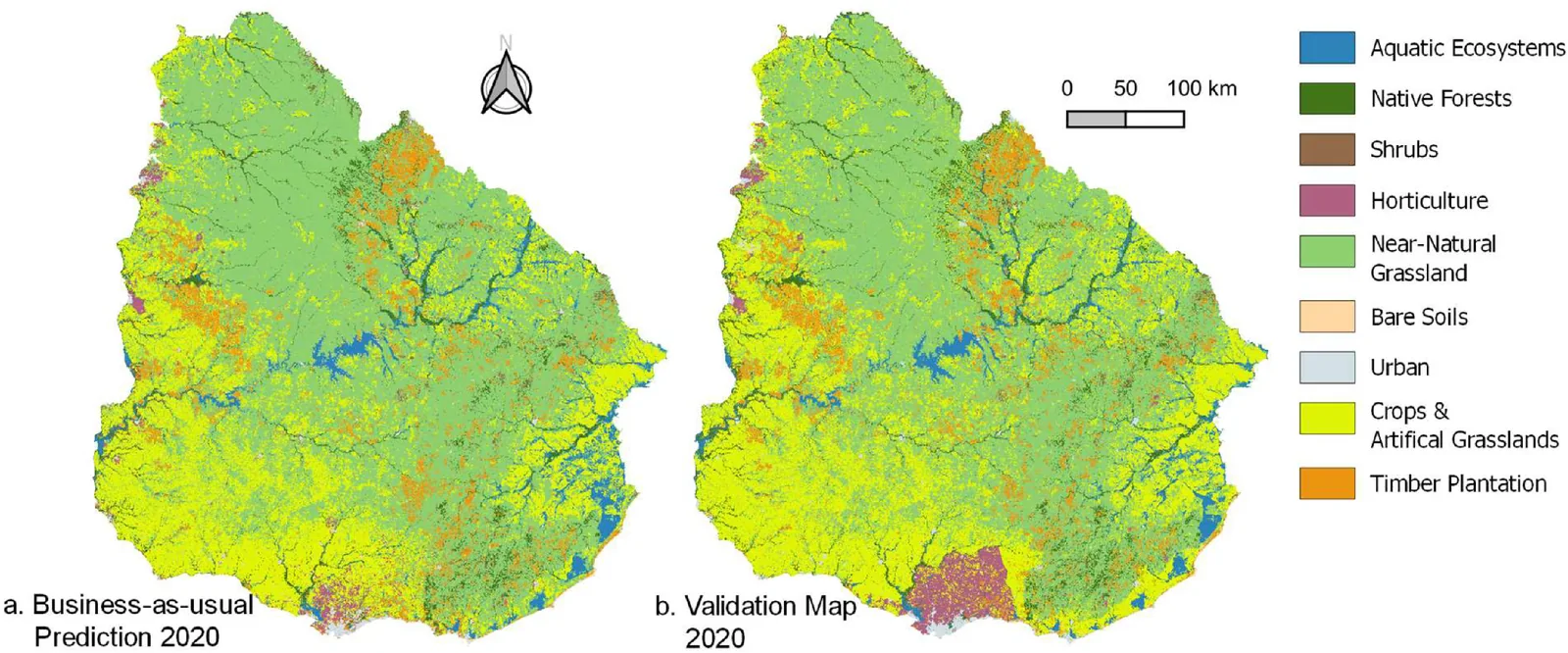
Predicted business-as-usual scenario and validation map for the year 2020.

Land-use maps produced by Uruguay's governmental institutions were used to model land-use change based on historical trends. These official datasets provide a reliable basis for policy-relevant scenario development. However, differences in classification methodologies, spatial resolution, and land-use classes required harmonization prior to modelling. The datasets were therefore reclassified, resampled, enhanced, and evaluated for classification accuracy to ensure temporal comparability.

Land-use maps from 2000 [12] and 2015 [13] were used to model historical land-use dynamics and to develop three land-use scenarios from 2015 onwards. Both datasets were derived from Landsat imagery with a spatial resolution of 30 × 30 m. Model validation was performed using the 2020 land-use map [14], which was primarily derived from Sentinel-2 imagery. The 2020 dataset was resampled from 10 × 10 m to 30 × 30 m resolution, and all datasets were converted from vector to raster format. Because no official accuracy assessment was available, classification accuracy was evaluated using stratified random sampling (570 validation points), resulting in overall accuracies of 85 % (2000), 66 % (2015), and 80 % (2020). All preprocessing steps were carried out in QGIS v3.22.4 [15]. All three original land-use maps were aggregated and reclassified into nine land-use classes that were consistently represented across all datasets: i) aquatic ecosystems (AE), ii) native forests (NF), iii) shrubs (S), iv) horticulture (Hc), v) near-natural grasslands (NNG), vi) bare Soils (BS), vii) urban (U), viii) crops & artificial grassland (CAG) ands ix) timber plantations (TP). Contrary to the increase in TP and NF and the strong decrease in NNG expected from the literature and the Uruguayan institutions [12], inconsistencies in land-use area estimates between the 2000, 2015, and 2020 datasets were observed. To enhance data comparability, we augmented the historical maps with a pixel mask from 2020. The harmonization approach was based on the assumption that areas classified as near-natural grassland (NNG) in the 2020 dataset were already present in the earlier land-use maps but had been underestimated because of methodological differences in land-use classification.

*Details Data Sources and Preprocessing:* We downloaded data, if not otherwise specified, from the website of the Ministry of Housing, Land Management and Environment [16]. Using Uruguay's conservative outline, we mapped our land-use maps, driver variables and land panning areas. The northeastern disputed territory near the village of Masoller, bordering Brazil, was excluded because complete land-use data for the Uruguayan portion of this region were unavailable. We re-projected all geospatial information in datum WGS84 and UTM-21S coordinate system (EPSG: 32,721). The pixel size of all files is 30 × 30 m; if not otherwise described, we pre-processed data QGIS v.3.22.14-Białowieża [15]. We converted all original files to the Idrisi Raster Format, mandatory for modelling with the Land Change Modeler (LCM) in TerrSet v.2020 [11].

*Reclassification to Nine Land Classes:* The 2020 map comprises 27 land-use classes, which we reclassified to 9. Similarly, we resampled the 17 and 16 classes of 2000 and 2015 to 9, respectively. We specify the merged classes in Table 5. Considering our study's focus on the impact of land-use changes on aquatic ecosystems, we combined the land-use classes of artificial water, natural water, and flooding areas in all three maps. The 2020 map classified commercial cropland classes into a more differentiated set of seven classes, which we merged into 'crops & artificial grasslands' (CAG). In 2000 and 2015, only two and one commercial cropland classes were defined, respectively. 'Regenerated grassland' is introduced as a new land-use class in the 2020 map encompassing areas with modifications, pastures, or stubbles, which we merged into CAG. The class 'near-natural grasslands' (NNG) includes unmodified grasslands with assumed livestock breeding in all three maps, reflecting the common land management practice of grasslands in Uruguay [12]. Urban areas were differentiated in more detail in the 2000 and 2015 maps than in the 2020 map. However, areas associated with horticulture practices were more detailed in the 2020 map. We merged all classes of 2020 related to horticulture, including orchards such as olive, citrus, and others, into the 'horticulture' class. Moreover, we merged the land class described in 2020 as horticulture zone into horticulture. This land class is described as an area that may include open soil or grassland but is devoted to horticultural practices. For the 2000 and 2015 maps, we merged the classes 'fruit trees' and 'irrigated and rainfed cropland smaller than five hectares' into the 'horticulture' class. Although the presence of palm groves in all three maps, we decided to merge palms into NNG because of their insignificant share (0.09 %) of the total area and their association with the savannah ecosystem.

**Table 5:** Reclassified Land cover maps for the years 2000, 2015 and 2020 [16]. If in column years & is used the class names and description are identical. If a / is used the term in class name and description differs.

Years	Class name in original map (Spanish)	Description English	Land class	ID
2000 & 2015 / 2020	Aguas artificiales / Represas de riego	Artificial Water / Water bodies, Artificial Hydroelectric dams and water reservoirs for human and other supply.	AE	1
2000 & 2015 / 2020	Areas desnudas / Playas, Dunas	Naked Areas / All types of beaches and dunes.	BS	6
2000 & 2015 & 2020	Canteras, areneras, minas a cielo abierto	Quarries, sand pits, opencast mines, Mines	BS	6
2000 & 2015 & 2020	Aguas naturales	Natural water	AE	1
2000 & 2015 & 2020	Arbustos	Shrubs	S	3
2000 & 2015 / 2020	Areas naturales inundadas / Bañados, humedales	Flooding natural areas / Wetlands at or near recurrent or sustained flooding or saturation at or near the surface.	AE	1
2000 & 2015 / 2020	Cultivos regados y de secano < 4–5 has / Horticultura	Irrigated and rainfed crops < 4–5 ha/ Horticulture	Hc	4
2000	Cultivos regados > 4–5 has	Irrigated crops > 4–5 ha	CAG	8
2000	Cultivos de secano > 4–5 has	Rainfed crops > 4–5 ha	CAG	8
2015	Cultivos regados y de secano < 4–5 ha	Irrigated and rainfed cropland < 4–5 ha	CAG	8
2020	Pivotes	Irrigated Crops with centre-pivot	CAG	8
2020	Mezcla CN, pasturas y rastrojos	Mixture of natural field, pasture and stubble areas that include sown grassland forages, associated with dairy farming.	CAG	8
2020	Rastrojos de cultivos cerealeros de invierno	Winter cereal crop stubble	CAG	8
2020	Rastrojos de Arroz & Cultivo de Arroz	Rice Stubble & Rice Cultivation	CAG	8
2020	Rastrojos de Pasturas	Pasture stubble	CAG	8
2020	Agricultura de secano, verano, grano seco y otros propósitos	Rainfed agriculture, summer, dry grain and other purposes	CAG	8
2020	Campo Natural regenerado	Regenerated grassland heavily modified natural grassland, introduced pastures and old stubble.	CAG	8
2020	Caña de Azucar	Sugar cane	CAG	8
2000 / 2015	Equipamiento Urbano	Urban equipment	U	7
2000 / 2015	Areas Urbanas dispersas	Scattered Urban areas	U	7
2000 / 2015	Area Urbana	Scattered Urban area	U	7
2020	Zona Uizada	Urbanized Zones, Urban areas (parks, industries, airports, sports fields, infrastructure, horticultural crops can be included)	U	7
2000 & 2015 / 2020	Frutales / Otros frutales	Fruit trees / Other fruit trees	Hc	4
2020	Zona Hortifrutiviticola	Horticulture Zone, areas devoted to fruit growing and vineyards, which may also include other uses on uncovered soils or with various herbaceous.	Hc	4
2020	Olivares	Olive plantations occasionally may include trees of other species.	Hc	4
2020	Citrus	Citrus trees	Hc	4
2000 & 2015 / 2020	Herbaceo Natural / Campo Natural	Natural grassland with little anthropogenic modification	NNG	5
2000 & 2015 / 2020	Monte nativo / Bosque Nativo	Native Forest / Native Forest	NF	2
2000 & 2015 / 2020	Palmares / Campo Natural y Palmares	Palm groves / Natural Grassland and palms	NNG	5
2000 & 2015 / 2020	Plantacion forestal / Bosque Plantado	Forestry planting / Planted forests	TP	9
2020	Bosque Plantado (nuevo, rebrote, tala)	Planted forests (new, harvested, regrowth)	TP	9

*Refinement of Historical Land-use Maps:* When comparing 2000, 2015, and 2020 land-use maps, we expected the total area of NF and TP to show a more stable increase, while NNG would exhibit a steeper decrease. However, our observations indicate discrepancies. TP’s total area percentage changed from 4 % (2000) to 8 % (2015) and 5 % (2020). NF, expected to increase, declined from 6 % (2000) to 5 % (2015) and held steady at 5 % in 2020. Against expectations, NNG, anticipated to decline steeply, showed percentages of 66 %, 53 %, and 51 % in 2000, 2015, and 2020, respectively. We aimed to accurately predict transitions related to grassland conversion, particularly in the context of timber expansion. However, the data before improvement did not strongly reflect these historical trends. We attributed discrepancies between observed and expected land changes for certain classes to methodological differences in classification. The two historical maps were classified using a segmentation method [12], resulting in an underestimation of grassland pixels as they were filtered out amidst anthropogenic land-use patches (Fig. 4, Fig. 5). We acknowledged the use of recent products (Sentinel 2, Landsat 8 data, survey data) for the 2020 classification and its regular updates by the Ministry of Housing, Land Management, and Environment, making it a reliable source for our data refinement. In Uruguay, we assumed grassland to be the predominating landscape, with anthropogenic changes leading to transitions. The natural succession involves a reduction of grasslands transitioning to shrubland, then native forest (NF). Where near-natural grasslands (NNG) were identified in 2020, we inferred its presence in 2000 and 2015. Further, if 'regenerated grasslands' were identified in timber plantations (TP) in 2020, indicating agrochemical runoff and silvopasture practices, we assumed a similar intensive use of areas between timber plantations in the historical maps, as it has been a common practice

**Fig. 4:**
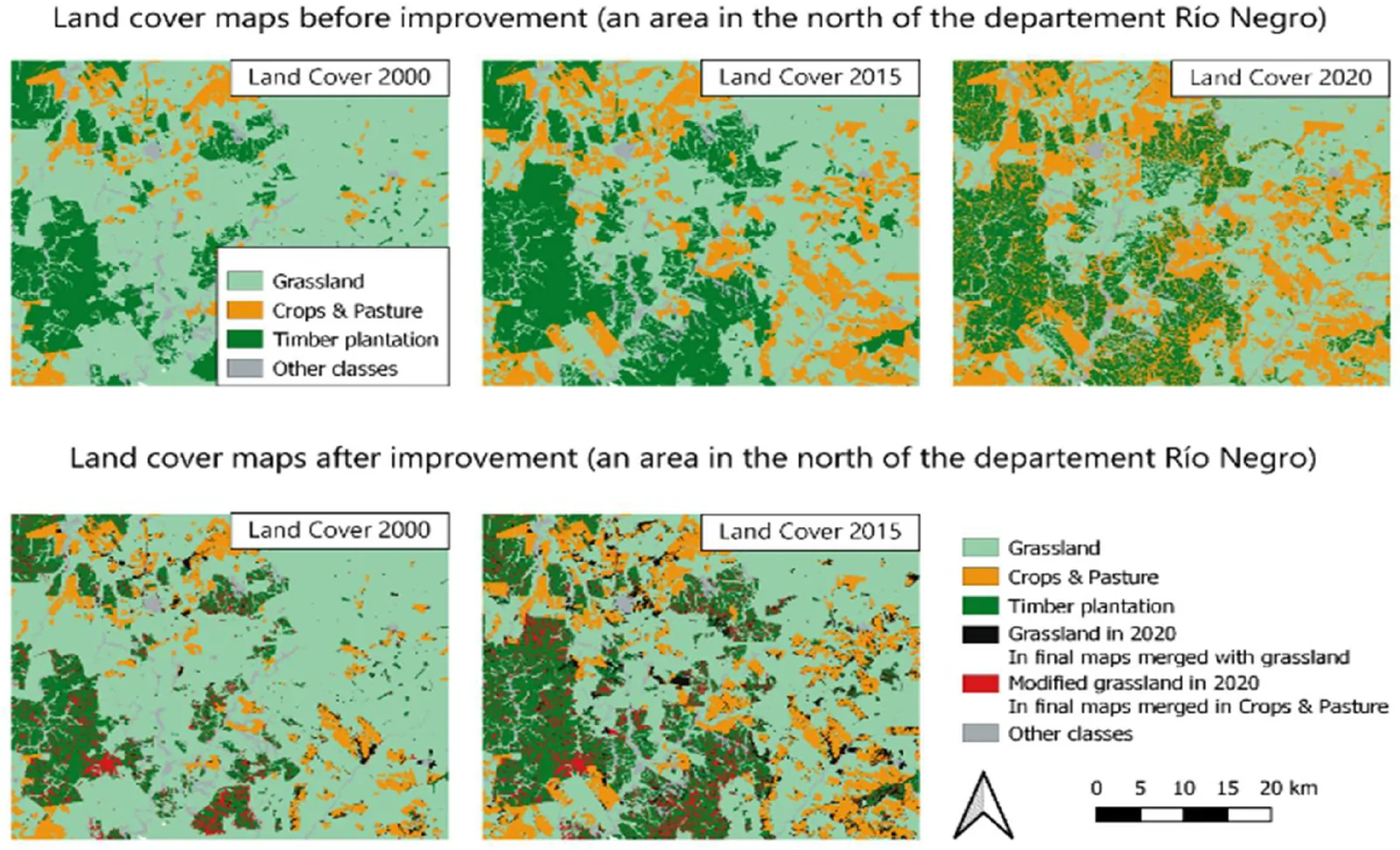
Subarea with near-natural grassland and agriculture before and after the data enhancement.

**Fig. 5:**
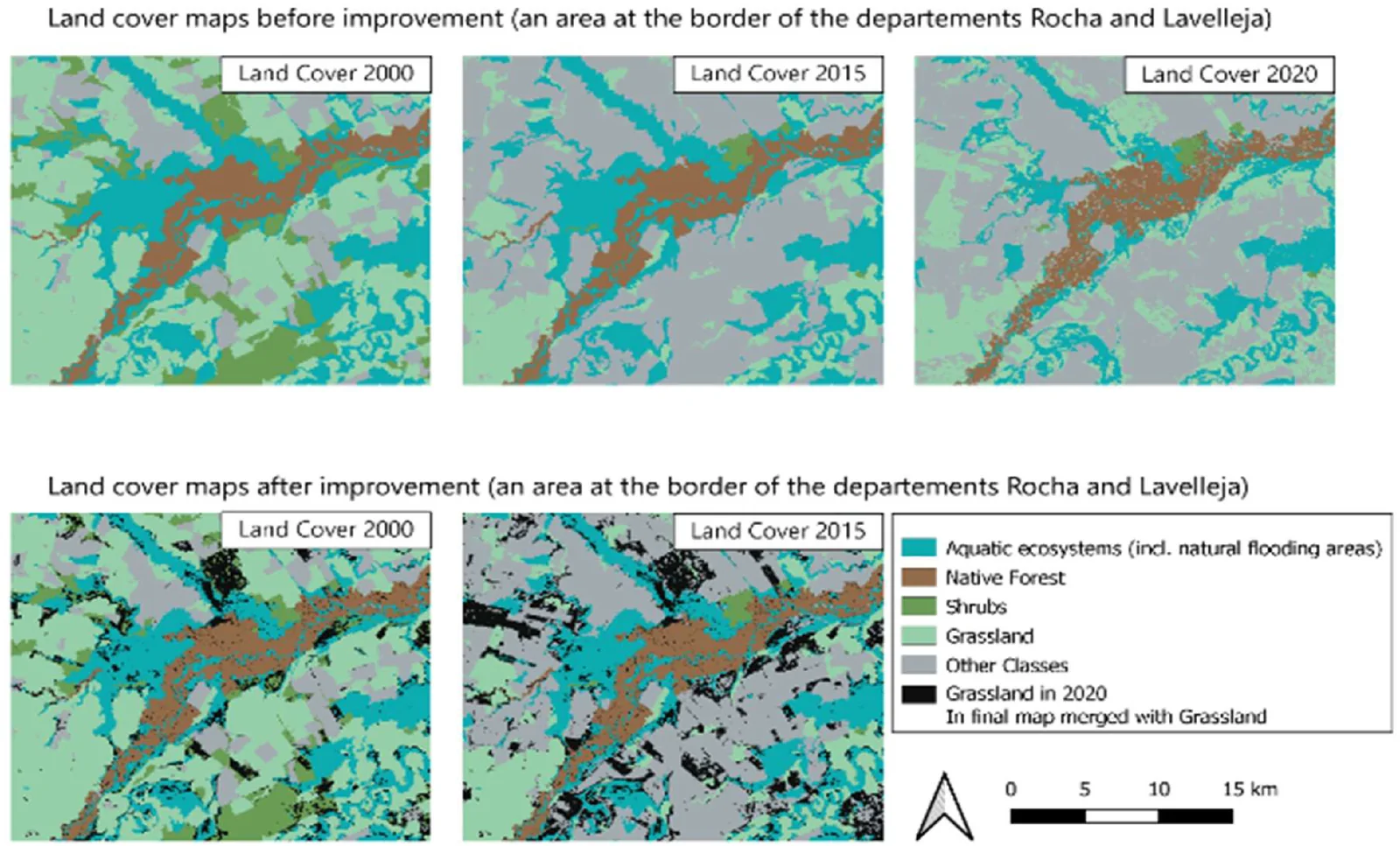
Subarea with native forest before and after data enhancement.

To overcome the different classification techniques in the historical and the validation maps, we enhanced the historical maps by applying a 2020 mask of NNG pixels by reclassifying intersecting pixels of all other classes to NNG in both historical maps. Moreover, we applied a mask of the class ``regenerated grasslands'' from the original 2020 map. We reclassified it as CAG when it intersected with TP pixels in both historical maps (Fig. 4, Fig. 5). The enhancement procedure was implemented using Python scripts.

Total area changes after improving the historical maps stand out for NNGs with an increase of 5 % (2000) and 7.6 % (2015), a decrease for NFs of 1.8 % (2000) and 1.2 % (2015), a decrease in CAG of 0.8 % (2000) and 2.7 % (2015) and a decrease in TPs of 1.4 % (2000) and 2.6 % (2015). Proportional class differences were significant for TPs with >30 % (see Table 6 area changes per class in detail). After refining the historical maps, the land changes from 2000 to 2015 and 2020 align with expectations. The total area of NNG shows a steady decline of 71 % (2000), 60 % (2015), and 50.7 % (2020). NFs declined from 2000 to 2015 (4.2 % to 3.7 %) but reached 4.6 % in 2020. TPs increase from 3 % (2000) to 6 % (2020) (Table 7).

**Table 6:** Land-use class net area changes (%) and proportional difference for class area after map enhancement (%) of Uruguay for the years 2000 and 2015.

	2000		2015	
	Net area change (%)	Proportion class area changes of total area (%)	Net area changes (%)	Proportion class area changes of total area (%)
Aquatic Ecosystems	−20	−0.92	−22	−1.10
Native Forests	−30	−1.76	−24	−1.15
Shrubs	−14	−0.19	−3	−0.04
Horticulture	−6	−0.05	−5	−0.06
Near-Natural Grasslands	8	5.09	15	7.64
Bare Soils	−25	−0.07	−31	−0.08
Urban	−3	−0.02	−4	−0.03
Crops & Artificial Grassland	−5	−0.72	−10	−2.65
Timber Plantation	−32	−1.37	−33	−2.59

**Table 7:** Percent of total area (%) of Uruguay by land-use classes for the years 2000, 2015 before and after map enhancement and for the year 2020.

Land-use classes	2000		2015		2020
	Total area (%) before enhancement	Total area (%) after enhancement	Total area (%) before enhancement	Total area (%) after enhancement	Total area (%)
Aquatic Ecosystems	4.6	3.7	4.9	3.8	3.5
Native Forests	6.0	4.2	4.8	3.7	4.6
Shrubs	1.4	1.2	1.3	1.2	0.9
Horticulture	0.9	0.9	1.1	1.1	2.4
Near-Natural Grasslands	66.1	71.2	52.6	60.3	50.7
Bare Soils	0.3	0.2	0.3	0.2	0.1
Urban	0.7	0.7	0.8	0.8	0.9
Crops & Artificial Grassland	15.7	15.0	26.3	23.7	30.8
Timber Plantation	4.3	3.0	7.9	5.3	6.1

*Accuracy Assessment*: From Uruguayan institutions, no accuracies are reported for either of the three classification maps. We conducted an accuracy assessment for the 2000 and 2020 maps by creating random stratified samples of 570 points. Following [17], we created a confusion matrix, with an overall accuracy of 85 % for 2000 (Table 8) and 80 % for 2020 (Table 9). We utilised QGIS 3.22.4 for all steps [15]. An overall accuracy of 66 % for the 2015 land-use map was calculated (see [10]), similarly as described before.

**Table 8:** Accuracy assessment land-use map 2000.

		1	2	3	4	5	6	7	8	9	Sum of pixels	User's accuracy (%)
		AE	NF	S	Hc	NNG	BS	U	CAG	TP		
1	AE	42	2								44	95
2	NF	4	20	2		2					28	71
3	S			11					1		12	92
4	Hc				7				1		8	88
5	NNG	8	4	20	6	296			18	2	354	84
6	BS						16				16	100
7	U				2	1		9	1		13	69
8	CAG			1		7			67		75	89
9	TP		1			2				17	20	85
Sum of pixels		54	27	34	15	308	16	9	88	19		
Producer's accuracy (%)		78	74	32	47	96	100	100	76	89	Overall accuracy	85.09

***Note:*** Aquatic ecosystems (AE); native forests (NF); shrubs (S); horticulture (Hc); near-natural grasslands (NNG); bare soils (BS); urban (U); crops and artificial grassland (CAG); timber plantation (TP).

**Table 9:** Accuracy assessment land-use map 2020.

		1	2	3	4	5	6	7	8	9	Sum of pixels	User's accuracy (%)
		AE	NF	S	Hc	NNG	BS	U	CAG	TP		
1	AE	31									31	100
2	NF		25	2		2					29	86
3	S			9					1		10	90
4	Hc				15	2			1		18	83
5	NNG	12	2	16	1	209			17	5	262	80
6	BS						19				19	100
7	U			1	1			10			12	83
8	CAG	6	1	5	4	27			111	4	158	70
9	TP	1							1	29	31	94
Sum of pixels		50	28	33	21	240	19	10	131	38		
Producer's accuracy (%)		62	89	27	71	87	100	100	85	76	Overall accuracy	80.35

***Note:*** Aquatic ecosystems (AE); native forests (NF); shrubs (S); horticulture (Hc); near-natural grasslands (NNG); bare soils (BS); urban (U); crops and artificial grassland (CAG); timber plantation (TP).

*Details on Explanatory Variables:* We selected 20 variables for their explanatory power in both persistent and transitioning pixels following MLP recommendations. Where these criteria were met, variables with similar spatial patterns or representing the same underlying processes were combined, as recommended to reduce the number of explanatory variables and improve model parsimony. Unless stated otherwise, all datasets were converted from vector to raster format. For all variables with “dist” preceded, we calculated Euclidean distance in TerrSet v.2020 [11].

Accessibility variables representing transport infrastructure and landscape exposure were selected because of their expected influence on land-use transition probabilities. Specifically, we considered *dist_roads_big* for major roads and *dist_roads_all* the entire road network. *Dist_ports* accounts for cargo-movement ports vital in agricultural transportation [16]. We derived *dist_U* as a binary raster from the 2000 land-use map in TerrSet v.2020 [11], illustrating proximity to Urban areas and its relevance for infrastructure and employment. *Dist_water* encompasses navigable waterways [16] proximity to waterbodies, and reflects water reservoirs, irrigation potential, and transportation through navigable waterways.

The topographic variables, *slope_percent* and *DEM*, could explain the suitability of management practices. Uruguay's elevation and slope exhibit low variability. We calculated with *DEM* [16] the slope percentage in TerrSet v.2020 [11]. *Temp_season* and *precip_season*, derived from BIO 4 and 15 from Fick and Hijmans [18], provide condensed information on temperature seasonality and precipitation seasonality coefficients of variation, respectively. All datasets were resampled to a spatial resolution of 30 × 30 m using bilinear interpolation in ArcMap 10.7. Binary raster layers were then generated for each persistent and transitioning land-use class in the 2000 land-use map involved in the nine modelled transitions (*dist_NF, dist_S, dist_AE, dist_NNG, dist_CAG, dist_TP*).

The variable *dist_SNAP_IBA* was derived by combining the National System of Protected Areas (SNAP), national protected areas, and Important Bird and Biodiversity Areas (IBA) [16] to capture the influence of nature conservation and areas of conservation interest. The variable *pop_dens* contains per-pixel information on population density in 2015 [19]. We resampled the data to a 30 × 30 m pixel size using the bilinear interpolation method in ArcMap 10.7. The variable dist_*modis_fire* holds information on fires from 2000 to 2011 with a 90 % certainty [20]. Fires in the Uruguayan context can be anthropogenic in pasture or forestry sites, or natural in savanna systems. *Coneat_all* was used as a soil variable, representing an index combining land costs and management recommendations (ranging from 0 to 206). Coneat_2789, a subset prioritizing soil groups for timber plantations, including sandy to loam-silt textured soils, was only employed to explain timber expansion. We created the variable *dist_saw_nursery* [16] by combining sawmills and tree nurseries strategically placed in timber plantation areas to capture economic influences and factors affecting plantation site conditions. The pulp mills variable was omitted, as the two major factories in 2022 are located at ports, potentially duplicating information for the model.

The 2022 livestock density data is provided at the minor administrative level [21]. The animal per hectare ratio at the administrative level was calculated. The *cattle_dens* variable, representing cattle density with a total population of 11.5 million animals and an average of 0.7 animals per hectare per administrative level, was included for all transitions. Silvopasture practices, primarily involving cattle, are prevalent within native forests and timber plantations. Dairy cows, the second-largest livestock category with 6.8 million animals, are exclusively distributed around Montevideo, averaging 0.1 animals per hectare per administrative level. Sheep, the third-largest livestock category, total 6.1 million animals with a median of 0.3 animals per hectare per administrative level. The latter livestock are included as *sheep_dens* and *dairy_dens* specifically for CAG related transitions. Horses, pigs, and goats are among the less relevant livestock in Uruguay, and we did not further consider them in our study.

*Variables for Prediction:* Using the TerrSet v.2020 Macro Modeler tool [11], combined constraint maps with a value of 0.01 for the three scenarios were generated, employing overlay. In addition, incentive maps were created using the same method, with a value of 4, specifically for the multifunctional landscape (MFL) and grassland conservation (GC) scenarios. For the business-as-usual (BAU) scenario, we utilized areas of SNAP, Ramsar wetlands, and palm forests, extracted as a binary raster from the original 2015 land-use map before reclassification. Incentive and constraint areas for the MFL scenario included SNAP, Ramsar wetlands, and palm forests, with an additional 90 m buffer around these zones. A 90 m buffer around native forests, aquatic ecosystems, and urban land-use classes was also calculated in 2015. Additionally, a 90 m buffer around schools was calculated. For the constraint and incentive areas in the GC scenario, we employed the same areas as in the MFL scenario but calculated each buffer with a 180 m radius. Additionally, an area for grassland protection for the constraint area was defined for the GC scenario. Buffer distances of 90 m and 180 m were selected to reflect empirically informed mitigation distances and to test precautionary spatial planning sensitivity within the scenario framework.

Our aim was to find an area with the largest and least fragmented grassland areas, where the impact of intensive use is minimised and the soils have comparatively low CONEAT values, indicating low productivity and low land costs. To achieve this, a threefold approach was applied. First, to find the largest, less fragmented grassland areas, we created a mask of pixels that were always grassland in the land-use maps of 2000, 2015, and 2020 (Fig. 6b). The largest permanent grassland area is located in the north of Uruguay above the Rincón del Bonete lake, where fragmentation due to anthropogenic land-use is minimal. Second, scanning the CONEAT description, we identified a threshold for soils with an index below 80, indicating limited agricultural use and potential suitability for grassland or high-maintenance agriculture. Although the distribution of CONEAT soil up to an index of 79 is less clear compared to permanent grasslands, we observed frequent pixels meeting our threshold from the area in the north of Uruguay above the Rincón del Bonete lake (Fig. 6a).

**Fig. 6:**
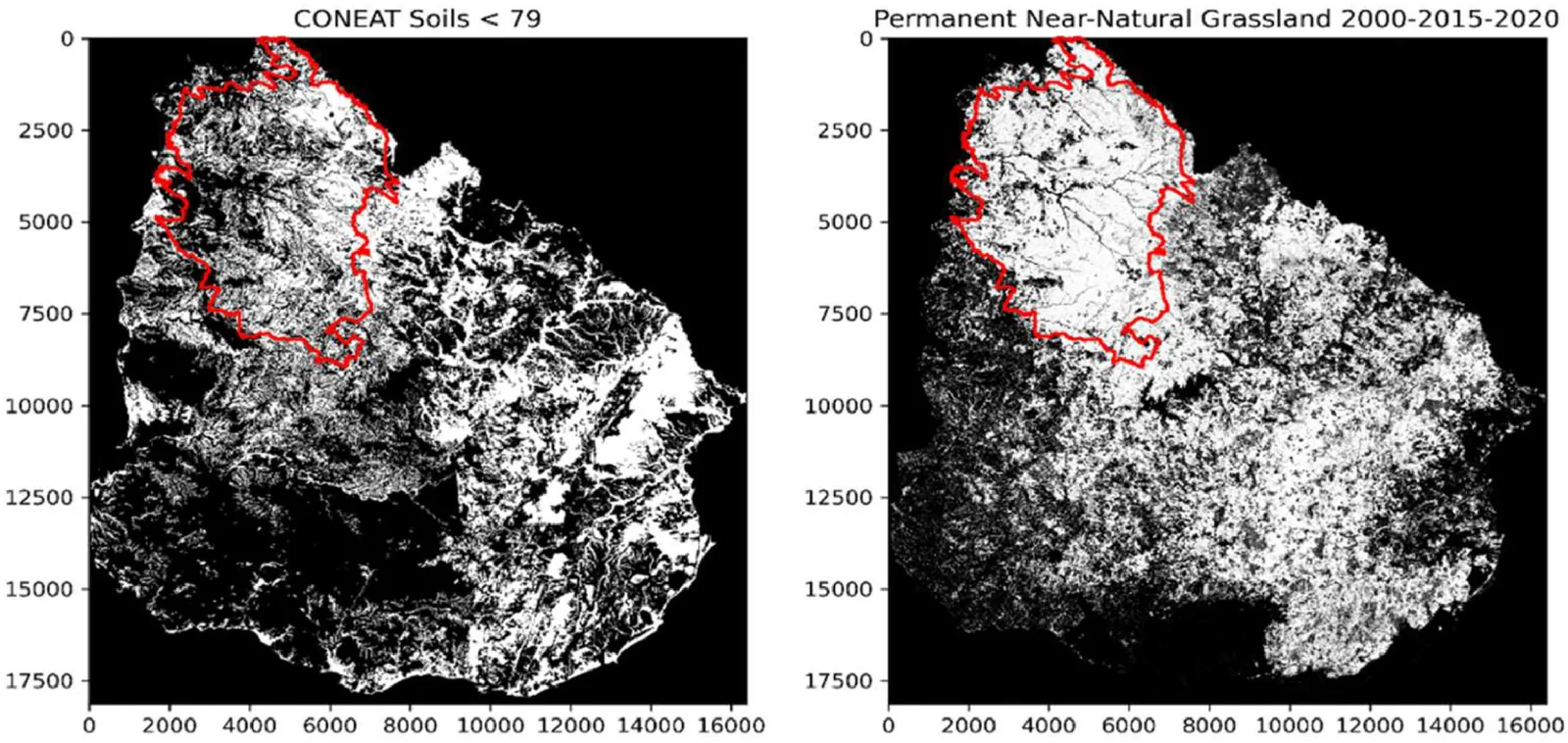
Distribution of areas with a) CONEAT soil productivity index up to 79, indicating a low management practice and b) Permanent near-natural grasslands consistent in the years 2000, 2015 and 2020, red line represents the identified old-growth grassland zone.

Third, we use livestock density per hectare (defined above in Details on Explanatory Variables) as a proxy for how intensive grasslands are used, with a focus on cattle, dairy cows, and sheep as significant livestock. The high proportion of sheep among cattle is a traditional grassland management practice on native grasslands [9]. Grazing by sheep can enhance grasslands, as they do not selectively graze, allowing for diverse species dispersal. Maintaining low grass heights through sheep grazing creates a habitat for bird species dependent on short grasses. Hence, high sheep density as a key indicator for diverse grasslands was utilized. Fig. 7 illustrates the distribution of the three livestock densities using their natural Jenks breaks. In the code for this analysis, we also tested the quantiles against the natural Jenks breaks value distribution, but natural Jenks breaks are contrary to quantiles sensitive to outliers and capture inherent data patterns. The area with the two highest natural Jenks breaks, with 0.54–1.71 sheep per hectare, as our old-growth grassland zone for conservation was selected (Fig. 7). This area meets the criteria of high grassland persistence, a low soil productivity index, and the highest sheep density, while minimizing pressure from dairy cows and cattle.

**Fig. 7:**
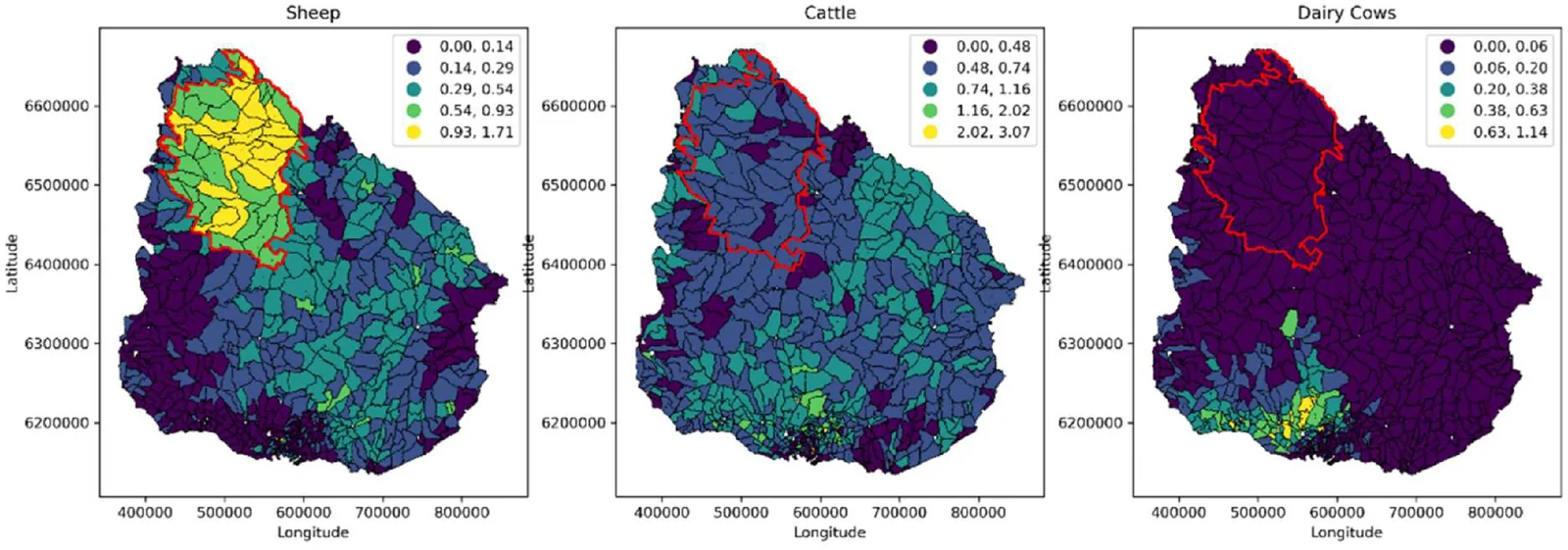
Livestock density per hectare aggregated at the minor administrative level for sheep, cattle and dairy cows. The data is displayed in five natural Jenks breaks, which provide sensitivity to outliers and capture inherent data patterns. The first highest natural Jenks breaks of sheep are chosen as proxy for traditional grassland management practices, red line represents the identified old-growth grassland zone.

### 4.2 Modelling of historical land-use change and prediction of scenarios

In this section we provide information on applying Land Change Modeler (LCM) for transition analysis; on the Multi-Layer Perceptron (MLP) neural network trained on historical land-use changes; on the Markov Chain model used for probability estimation of transitions and on three scenarios implemented with constraint and incentive maps.

The change analysis in LCM offers pixel-per-pixel land-use class changes generated as graphs representing gains, losses, and net change between 2000 and 2015. Contributions to net changes of each land class and maps of persistence and specific transitions, expressed valuable details to identify essential transitions. A threshold of at least 1000 ha absolute change was used, to filter significant transitions for further modelling [3].

*Selection of Transitions and Explanatory Variables for Modelling*: The transition potential determines the likelihood of an area to undergo a transition. Transitions between land-use classes, can be ignored during further modelling; the ``from'' class is left as in the second land-use map (2015) until it is involved in another transition. Transitions in the later prediction modelling were ignored to reduce them to the most relevant transitions and to differentiate between the scenarios, which is a common modeler decision [3]. Transitions can be modelled individual or grouped for similar patterns of change and if they are explained by mutual drivers. We partly grouped transitions, but also compared the model accuracy with the individually modelled transitions. Drivers or explanatory variables are factors influencing or explaining land-use change. A total of 20 explanatory variables were prepared to evaluate their ability to explain land-use persistence and transitions; 10 are transition-specific and not used for all transition potentials (Hasan et al., 2020). Most variables were defined as static, whereas the distance-to-development variables were defined as dynamic, as we assume they change over time. Dynamic variables are recalculated by the MLP-Markov chain model over time [4].

*Multi-Layer Perceptron (MLP):* MLP is a machine learning algorithm utilising back-propagation, one of the most popular neural network models dealing with classifications and temporal change transitions [11]. The MLP model structure includes an input layer of land-use transition and explanatory variables, hidden layers for processing input data, and an output layer for the transition potential model and map output ([2]; Fig. 8). MLP is a highly capable neural network with interconnected nodes processing information through weighted inputs [1,7]. Its multi-layer structure enables it to handle non-linear data effectively, making it particularly adept at capturing and modelling complex non-linear relationships [3]. We use multi-layer perceptron (MLP) neural network as a powerful modelling algorithm, relying on the widely recognised back-propagation method, for handling classifications and temporal change transitions [3,6,7]. Thanks to its interconnected nodes and weighted inputs, the MLP neural network is adept at dealing with non-linear data. The multi-layered structure not only enhances its capability for non-linear data but also excels in capturing intricate relationships [3]. The MLP operates as a feedforward neural network, directing data sequentially from the input layers through hidden layers comprising computational nodes to the output layer. Nodes are linked through connections with assigned weights, and the network undergoes training by utilising a backpropagation algorithm. This involves iteratively propagating errors from the output layer to the input layers adjusting weights to minimise the disparity between observed and predicted outcomes. The weights across the multiple layers are in MLP computed with the non-linear sigmoidal function, eventually producing a output layer, outputted as transition potential [6].

**Fig. 8:**
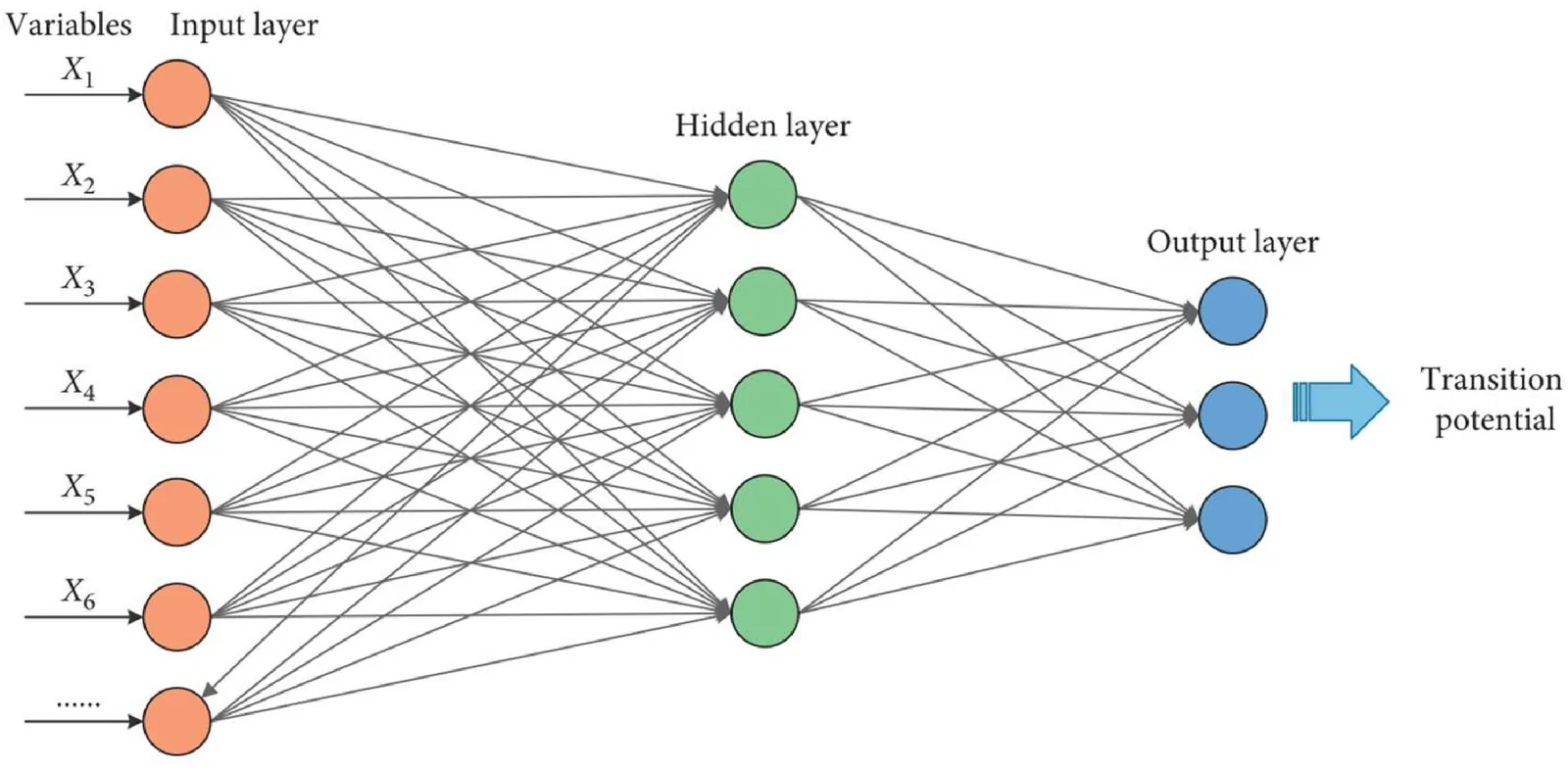
Multi-layer perceptron (MLP) model structure [2].

*Stopping Criteria, Detail on RMS, Learning rate:* Our approach incorporated a dynamic learning rate to balance convergence speed and precision. 10,000 iterations were conducted as the Root Mean Square (RMS) error curve demonstrated a consistent decrease and stability over this number of iterations, aligning with our approach of tracking the error curve for performance monitoring [22]. We use the default stopping training criteria, a Root Mean Square (RMS) error of 0.01, 10,000 iterations and an accuracy rate of 100 %. If either of these values is reached, the models training stops. The most critical parameter to tune in the MLP is the learning rate. In line with recommendations, we opted for the default parameter setting and implemented a dynamic training approach for the learning rate. During training, the performance of each transition model was assessed by analysing the error curve, where the RMS is ideally expected to decrease over time (asymptotic). The testing and training error RMS curves are ideally expected to decrease while being parallel, indicative of consistent performance evaluation. An increase or divergence in both error curves serves as a sign of overfitting, which compromises the generality of the network [11]. All models were allowed to run until 10,000 iterations, we observed an asymptotic decrease in the error curve leading to a stable low error value, denoting convergence. The MLP dynamically adjusts the start and end learning rates during this process. The start and end learning rates are respectively 0.01 and 0.001; when the training and testing RMS error oscillates substantially within the first 100 iterations, both learning rates are reduced by 50 %. Then, the modelling process restarts and finishes at 10,000 iterations when the RMS error graphs are more stable within the first 100 iterations - this dynamic adjustment of the learning rate balances convergence speed and precision.

*Accuracy Rate and Skill Measure:* After training the network with training pixels, the model automatically tests its ability to predict validation pixels. If not modelled separately, the performance of the MLP given for the group of transitions evaluated with two precision statistics: the accuracy rate and the skill measure. The accuracy rate reflects the model’s ability to predict validated classes. As Mas et al. [1] highlight, the skill measure is computed as the accuracy of transition prediction minus the accuracy expected by chance. Training and testing data is used for validating the prediction capability of each precision statistic. The accuracy rate reflects the test results after each iteration. It is crucial to note that some predictions in the validation test may occur by chance. For instance, in modelling a single transition, there's a 50 % chance of a correct answer purely by chance. As the number of transitions increases (grouped transitions), the likelihood of chance-driven correct predictions decreases. As defined in the manual, the expected accuracy rate considers the number of transitions and 'from' classes. Models with more transitions might have a lower accuracy rate because the chance of correctly predicting multiple transitions diminishes. It is recommended to only group a few transitions for MLP modelling. As Kumar and Agrawal [7] highlight, the skill measure is computed as the accuracy of transition prediction minus the accuracy expected by chance. It introduces a skill statistic ranging from −1 to +1, where 0 represents chance performance, 1 indicates perfect prediction and a negative value suggests performance worse than chance. This skill measure addresses the dependence of expected accuracy on the number of transition/persistence classes, providing a comprehensive evaluation of predictive capabilities.

*Reduction of Explanatory Variables:* We focused on the model’s output backwards stepwise variable elimination test, systematically removing variables with minimal impact on the skill of the model. Based on this approach, variables without explanatory power were removed, to reduce the risk of overfitting (lack of generality) and to potentially enhance the model's overall skill. In addition, highly correlated variables (Pearson r > 0.8) were excluded to reduce redundancy and improve model parsimony. Variable importance was assessed using the model's output test, in which each explanatory variable was individually held constant while model performance was evaluated, thereby simulating its exclusion from the model. To recognise potential outcome variations due to random weight initialisation, we prioritised the five most influential variables. Following the recommendations provided in the LCM and excluding Cramer's V test in TerrSet v.2020 [11], explanatory variables were evaluated using the model's output and skill assessments [3]. We employed sensitivity analyses, using two integrated tests from the model output to first reduce variables and second to interpret variable influence. MLP adjusts the number of hidden layer nodes to the number of variables. Expanding the number of hidden layers enables the MLP to tackle manifold data computation effectively [3]. While the number of explanatory variables is not explicitly limited for MLP, it is acknowledged that an increase in the number of variables can potentially lead to a rise in correlation among them. Notably, MLP exhibits the capability to handle correlated data, as it can capture the underlying structure in correlated variables during the learning process. We deliberately kept the number of variables within a reasonable range, a decision supported by the understanding that overly large networks with an abundance of variables can lead to overfitting and diminished generalisation performance. Guided by contextual relevance, with our selection on variables we aimed to enhance the model's ability to capture system dynamics. Thematic alignment ensured meaningful contributions to transition modelling. To further refine the variable selection, we employed the model outputs backwards stepwise constant forcing test, identifying variables with a minimal effect on skill measurement, reducing overfitting risks. In this test, also interactions among variables are explored. This systematic approach examines every possible pair of variables, determining their joint effect on skill when held constant and progresses by keeping additional variables constant until only one variable remains. In alignment with the outcomes of the backwards stepwise constant forcing test, identified variables were removed to improve skill, proceeding without these selected variables when both accuracy statistics showed improvement. Each transition model and generated map outputs were generated based on precision statistics, stopping when accuracy rates and skill measures ceased to improve after variable elimination using the Backwards Stepwise approach. The model’s output test “forcing a single independent variable to be constant” involves holding each variable individually constant while assessing the impact on the model's skill. This comprehensive analysis provides insights into each variable's isolated contribution to the model's predictive accuracy. We deemed this test valuable for understanding the individual significance of variables in our change prediction models. To evaluate the impact of variables of our final models, we used this test, where the weights of selected variables were held constant, akin to leaving the variable out of the model. This interpretation, resembling a feature elimination approach, provides insights into variable influence on the model. This approach allowed to us to bear in mind the potential variations in model outcomes due to random weight initialisation. As we observed a fluctuation of the influential variables for each training run, influenced by variations in overfitting and actual model quality, we only focused on the five most influential variables. The highest variation was observed among variables with a medium level of influence.

*Scenario Prediction and Land-use Planning with Markov Chain:* MLP derives as output a transition potential map for each modelled transition. Each transition potential map implicates the time-specific potential for the historical land class changes, based on the combination of influential driver variables. The transition potential map indicates a high-to-low rate of vulnerability to change with values between one and zero. To ensure specificity to modelled transitions, LCM masks pixels that do not match any given transitions ``from'' case. For instance, if the modelled transition is from NNG to CAP, values will exist only in pixels originally classified as NNG. The stochastic analysis process Markov chain is employed to quantify the expected change probabilities. This involves determining the probability for each land-use class to transition from 2015 to the prediction date, represented in a transition potential matrix. Each matrix cell denotes the probability of transitioning between different land-use classes, ensuring that the sum of probabilities for a class equals 1 and the persistence to remain the same land-use is in general the highest probability. The probability matrix was calculated for 2020 for validation and for 2030 for the final scenario predictions. The Markov chain component derives transition probabilities from observed land-use changes between 2000 and 2015. When projecting to 2020 and 2030, this approach assumes relative temporal stationarity of these transition probabilities. Structural shifts in market conditions, policy frameworks, or investment patterns may therefore introduce uncertainty into long-term projections. Notably, the transition potential matrix can be manually edited, as we did to account for less intensive anthropogenic land-use trends in the MLP and GC scenarios. Along the scenarios, we expanded the size of conservation areas, utilizing constraint maps to hinder cropland and timber expansion in sensitive areas, and incentive maps for promoting grassland regrowth within these areas. Incentive and constraint map values (0 to above 1) significantly impact the prediction map by multiplying with transition potential maps (see preparation of incentive and constraint maps in chapter on Variables for Prediction above). At any given time, numerous areas have the potential to change. Yet, a single prediction commits to one of many highly plausible scenarios, making the chances of accurate prediction quite slim compared to actual outcomes. The ``hard prediction'' is the allocation of the nine land classes through a competitive decision process, similar to multi-objective decision-making. Markov chain spatially allocates through all transition potential maps, along with constraint and incentive maps, predicting based on the 2015 base map and the transition probability matrix. We assess total area differences between scenarios with the hard prediction (land-use prediction map) and compare soft predictions (change potential maps), offering a comprehensive assessment of vulnerability to change for selected transitions. Values between one and zero indicate high to low change suitability, respectively.

### 4.3 Validation approach

The performance of the MLP-Markov chain model, trained on transition trends from 2000 to 2015, was evaluated for predicting land policy scenarios to 2030. The business-as-usual (BAU) scenario represents the expected outcome in reality, and its prediction for 2020 is tested against an actual map of the same year. The other scenarios involve notional conservation measures, which do not apply to the validation map. Model performance was assessed through visual comparison of the predicted and observed maps, complemented by an analysis of differences in land-use area. Predictive accuracy was evaluated using the kappa statistics K_no_, K_location_, K_locationStrata_ and K_standard_ [5,6,11]. These statistic components comprehensively evaluate both quantity and location of correctly classified cells, overcoming the limitations of the conventional kappa coefficient. K_no_ indicates the Kappa for no information, describing the overall accuracy of the predicted map compared to the actual map. K_location_ represents the kappa for grid cell level location, K_locationStrata_ for spatial accuracy for stratum level and K_standard_ is the kappa coefficient expressing the ratio of chance to correct assignments [6,7]. Kappa values are stratified into poor (below 0.40), fair to good (0.40 to 0.75), and excellent (above 0.75) [5]. Model validation was considered satisfactory when K_no_ exceeded 0.75, reflecting substantial agreement between predicted and observed land-use patterns according to established kappa interpretation thresholds. Moreover, we examine whether the MLP-Markov chain model effectively simulates anticipated land-use policies for our three scenarios. We aim to validate the predicted differences in land-use in Uruguay among the scenarios, with a specific focus on areas converted within the sensitive regions defined in the grassland conservation (GC) scenario. We refer to a subarea, showing the land-use policy difference along the scenarios in detail.

## Limitations

The dataset is based on harmonized national land-use maps for 2000, 2015, and 2020 that were originally produced using different classification methodologies and input data. Although the harmonization procedure improved temporal consistency, uncertainties associated with the original classifications may influence model calibration and simulated land-use transitions. The Land Change Modeler (LCM) applies a Multi-Layer Perceptron–Markov Chain approach in which future land-use change is estimated from historical transition probabilities. Consequently, the model assumes that the relationships between explanatory variables and land-use transitions remain stable over time and does not explicitly represent changes in policy implementation, market dynamics, or individual land-use decision-making processes. Furthermore, the scenario simulations are based on the selected explanatory variables and policy-specific constraint and incentive layers; therefore, alternative assumptions or additional socio-economic drivers may result in different spatial outcomes.

## Ethics Statement

The authors have read and follow the ethical requirements for publication in Data in Brief and confirm that the current work does not involve human subjects, animal experiments, or any data collected from social media platforms.

## Credit Author Statement

**Melanie Schweikart:** Methodology, formal analysis; investigation; data curation; visualization; writing—original draft; **Leonardo R. Ramírez:** Methodology; validation; formal analysis; investigation; supervision; data curation; writing—review and editing; **Ina Säumel:** Conceptualization; methodology; investigation; supervision; resources; funding acquisition; writing—review and editing.

## Data Availability

edoc-ServerDataset for land-use change scenario modelling in Uruguay using the Land Change Modeler (Original data).
